# Antigen-specific type 1 regulatory T cell responses shape immunity and disease tolerance in human malaria

**DOI:** 10.1172/JCI208046

**Published:** 2026-07-15

**Authors:** Leonie Brockmann

**Affiliations:** Human Biology-Microbiome-Quantum Research Center (WPI-Bio2Q), Keio University, Tokyo, Japan.

## Abstract

Type 1 regulatory (Tr1) T cells are a major source of IL-10–mediated immune regulation, yet their phenotypic definition and role in human disease remain incompletely understood. In this issue of the *Journal of Clinical Investigation*, Nideffer et al. provide insight into human Tr1 cells during pediatric *Plasmodium falciparum* (*Pf*) infection. The authors identified Tr1 cells as a major component of the malaria-specific CD4^+^ T cell response, producing both IL-10 and IFN-γ. They proposed that, in this context, Tr1 cells may be better identified by CD127 downregulation combined with CXCR6 expression than by other surface markers. Importantly, Tr1 cells exhibited suppressive function and were associated with reduced symptomatic disease but also with prolonged infection. Together, these findings refine current models of Tr1 cell identity and establish a more rigorous framework for marker validation using single-cell transcriptomics while highlighting the role of Tr1 cells in balancing immunity and immunopathology during infection.

## The unresolved identity of Tr1 cells

Type 1 regulatory (Tr1) T cells represent a major subset of IL-10–producing CD4^+^ T cells with potent immunosuppressive capacity. In contrast with Foxp3^+^ regulatory T (Treg) cells, Tr1 cells lack a lineage-defining transcription factor or stable phenotypic definition, complicating their identification in human disease. Whereas Foxp3^+^ Tregs follow a fixed developmental program, Tr1 cells appear to occupy a more flexible immunological space, currently defined primarily by suppressive function.

Since their initial description in the mid-1990s (1, 2), identification of surface markers CD49b and LAG-3 has represented a key advance (3), and Tr1 cells have been implicated in immune regulation across autoimmunity and transplantation (2–5). However, these markers are neither exclusive nor universally expressed, limiting their utility as cell-specific markers. Alternative combinations, including CCR5 and PD-1 coexpression, have been proposed (6), yet no consensus phenotype has emerged.

In parallel, transcriptional and functional studies have suggested that Tr1 identity reflects a conserved regulatory program. Across species and disease contexts, Tr1 cells share a unique signature characterized by IL-10 production, IFN-γ coexpression, and inhibitory receptor expression regulated by c-MAF and BLIMP-1 (7–10). This has led to a model in which Tr1 cells represent a regulatory program that can be adopted by conventional CD4^+^ T cells under defined inflammatory conditions (11, 12).

Recent single-cell analyses have added further resolution, revealing substantial heterogeneity within Tr1-like populations. Distinct Tr1 states, including naive-like, effector, memory, and activated subsets, emerge within antigen-specific CD4^+^ T cells, indicating that Tr1 identity is dynamically structured along differentiation trajectories (13). In malaria, single-cell studies further show partial overlap between Tr1-like transcriptional programs and canonical Th1 effector states (14), suggesting closer coupling between regulatory and effector fates than previously appreciated. Importantly, surface marker–defined Tr1 populations show only partial overlap with transcriptionally defined clusters, underscoring the limitations of conventional gating strategies in capturing underlying biology (13, 14), raising the possibility that Tr1 cells have been misidentified or underreported across studies, especially in humans.

## A dominant Tr1 response in human malaria

**Figure 1:**
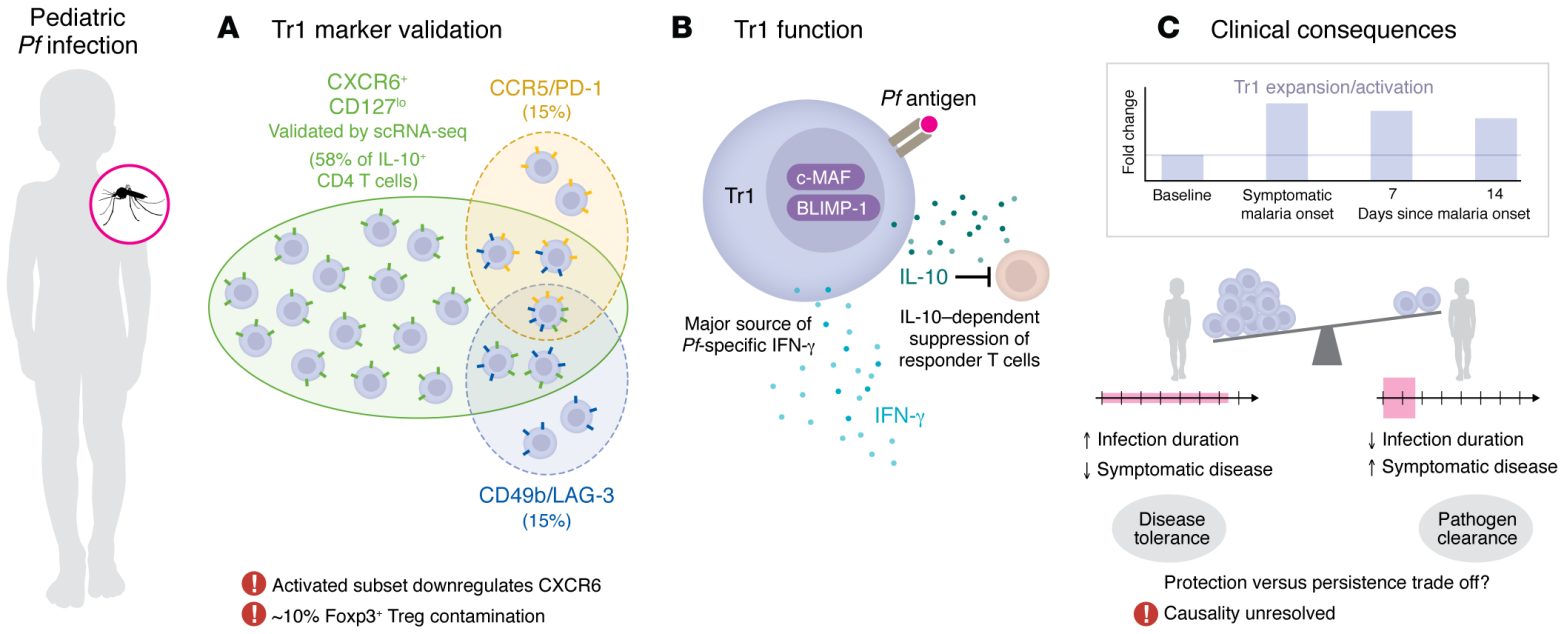
Tr1 cells in human malaria: identification, function, and clinical trade off. Nideffer et al. (15) examined the malaria-specific CD4^+^ T cell response in a pediatric population infected with *P*. *falciparum* (*Pf*). (**A**) They identified the combination of CD127^lo^ and CXCR6^+^ as markers that captured a larger fraction of IL-10^+^ memory CD4^+^ T cells (defined as Tr1 cells) than conventional CD49b/LAG-3 or CCR5/PD-1. However, activated Tr1 cells downregulated CXCR6 and were missed by the CD127low/CXCR6^+^ combination, and approximately 10% of the cells inside the gate were Foxp3^+^ Tregs. The phenotype was validated by scRNA-seq, setting a new standard for Tr1 marker identification. (**B**) Tr1 cells expanded during symptomatic malaria and produced more IL-10 and IFN-γ upon infection. They were a major source of *Pf-*specific IL-10 and IFN-γ (not Th1 cells) and suppressed responder T cell proliferation in an IL-10–dependent manner. (**C**) A longitudinal study in a high-risk pediatric population found that Tr1 expansion and activation coincided with the onset of symptomatic malaria. Higher Tr1 frequencies were associated with reduced symptomatic malaria but also with prolonged infection, a potential trade-off between disease tolerance and parasite clearance whose causality remains unresolved.

In this issue, Nideffer et al. (15) define the functional role of human Tr1 cells during pediatric *Plasmodium falciparum* (*Pf*) infection. By integrating phenotypic enrichment with single-cell transcriptomic datasets, the authors demonstrated that Tr1 cells were a major component of the malaria-specific CD4^+^ T cell response in this pediatric cohort (Figure 1A), adding to growing evidence that Tr1 cells and regulatory programs are a core feature of antiparasitic immunity (7, 13, 16–18).

Beyond abundance and expansion, Tr1 cells identified by Nideffer et al. contributed substantially to the antigen-specific response. They represented a prominent source of IL-10 following stimulation with *Plasmodium-*infected red blood cells and contributed considerably to IFN-γ production in an antigen-specific context (Figure 1B). Conventionally defined Th1 cells from the same patients produced little IFN-γ upon *Pf*-specific restimulation, suggesting that Tr1 cells, rather than classical Th1 cells, are the major source of malaria-specific IFN-γ in this setting. While IFN-γ and IL-10 coproduction has been observed in malaria-specific CD4^+^ T cell responses (7, 16, 19), Nideffer et al. directly linked this bifunctionality to a phenotypically defined Tr1 population adopting hybrid regulatory-effector states.

Furthermore, Tr1 cells also appeared linked to systemic immune regulation. Their frequency correlated with circulating IL-10, IFN-γ, granzyme A, and LAG-3, indicating participation in shaping the inflammatory environment during infection. Longitudinal analyses further showed Tr1 cell expansion during symptomatic malaria and associations with disease trajectory, consistent with a role for Tr1 cells in modulating clinical outcomes. However, whether Tr1 cells actively enforce disease tolerance or instead expand because of high antigen burden remains unresolved. Further investigation will be needed to establish if a causal relationship between Tr1 cells and reduced symptoms during malaria infection exists.

Importantly, higher Tr1 frequencies were associated with reduced symptomatic malaria, consistent with their established role in limiting immunopathology and promoting resolution of inflammation (20, 21), but also with prolonged infection, suggesting a trade-off between disease tolerance and parasite clearance (Figure 1C). In endemic regions, repeated *Pf* exposure often leads to asymptomatic infections, implying active immune regulation (22, 23). Nideffer et al. provide a potential explanation: pediatric patients with higher Tr1 frequencies had lower probability of developing symptomatic malaria. Beyond malaria, Tr1 cells have been implicated in other infectious diseases (7, 20, 21, 24), raising the question of whether the protective-but-persistent trade off observed here generalizes to other contexts.

## Redefining Tr1 cell markers

A key technical advance of the study is the identification of a CD127^lo^ CXCR6^+^ phenotype that improves enrichment of human Tr1 cells during *Pf* infection. Guided by single-cell transcriptional profiling (13), Nideffer et al. demonstrated that this population captured a larger fraction of IL-10–producing CD4^+^ T cells than previously used marker combinations CD49b/LAG-3 and CCR5/PD-1 (3, 6). This surface phenotype aligns with and extends prior transcriptional observations. Edwards et al. (7) independently identified downregulation of CD127 and expression of CXCR6 as part of the transcriptional signature of human Tr1 cells during *Pf* malaria, providing convergent validation, but without the phenotypic and functional analysis provided in the current study. Importantly, CD127^lo^ CXCR6^+^ cells exhibited canonical Tr1 features beyond IL-10 and IFN-γ production, namely, suppressive activity in vitro. This functional validation is critical, as suppressive capacity, beyond IL-10 production alone, remains a defining feature of bona fide Tr1 cells. Of note, suppression was assessed under polyclonal conditions due to technical constraints. Thus, whether Tr1 cells exert antigen-specific suppressive function during *Pf* infection remains an open question.

However, activated Tr1 cells, and some memory Tr1 cells, downregulated CXCR6, and thus a substantial fraction of IL-10–producing cells remained outside this gate, indicating that no single surface phenotype captures the full Tr1 spectrum in vivo. Partial overlap with CD127^lo^ CXCR6^+^ Foxp3^+^ Tregs further highlights ongoing specificity challenges, particularly in inflammatory contexts or in other tissues, where CD127 downregulation, and acquisition of CXCR6, is not unique to Tr1 cells (25).

Notably, Nideffer et al. observed limited overlap between Tr1 populations defined by different marker strategies. CD127^lo^ CXCR6^+^, CD49b/LAG-3, and CCR5/PD-1 identified largely distinct subsets despite all being enriched for IL-10 production, suggesting that current markers capture overlapping regulatory states rather than a single unified population. The study did not include a systematic single-cell comparison of these populations, which would help clarify whether they represent distinct subsets or different activation states of a shared program.

Collectively, these findings reinforce a central limitation of many human Tr1 studies: surface marker–based definitions incompletely capture transcriptionally and functionally defined regulatory programs. Thus, the CD127^lo^ CXCR6^+^ phenotype should be viewed as a context-specific enrichment strategy rather than a universal Tr1 marker, requiring validation across tissues and diseases. By validating their surface phenotype against single-cell transcriptomic data rather than IL-10 expression alone, Nideffer et al. establish a more rigorous benchmark for defining human Tr1 cells. These key themes — marker performance, functional validation, and clinical trade-off — are integrated in Figure 1.

## Open questions and future directions

Key questions remain. First, whether Tr1 cells represent a stable lineage or a dynamic functional state within broader CD4^+^ T cell differentiation trajectories remains debated. Second, the signals governing Tr1 differentiation and maintenance in vivo are still incompletely defined, including the roles of antigen load, inflammatory cues, and tissue-specific environments. Addressing these questions will require integrated approaches combining longitudinal single-cell profiling, clonal tracking, and functional perturbation systems. Finally, the therapeutic potential of Tr1 modulation remains an open area. While enhancing Tr1 responses may limit immunopathology, it may also impair pathogen clearance, highlighting the need for context-specific strategies.

Ultimately, Nideffer et al. establish Tr1 cells not as a niche regulatory subset but as a central part of the antigen-specific response to *Plasmodium falciparum*, prompting a reevaluation of how protective immunity and immunopathology are balanced in human malaria.

## Conflict of interest

The author is an inventor on a patent application (PCT/US2024/051612).

## Version 1 07/15/2026

Electronic publication
